# 3-Methyl-3,4-dihydro-9*H*-carbazol-1(2*H*)-one

**DOI:** 10.1107/S1600536809028050

**Published:** 2009-07-22

**Authors:** A. Thomas Gunaseelan, K. Prabakaran, K. J. Rajendra Prasad, A. Thiruvalluvar, R. J. Butcher

**Affiliations:** a PG Research Department of Physics, Rajah Serfoji Government College (Autonomous), Thanjavur 613 005, Tamilnadu, India; b Department of Chemistry, Bharathiar University, Coimbatore 641 046, Tamilnadu, India; c Department of Chemistry, Howard University, 525 College Street NW, Washington, DC 20059, USA

## Abstract

In the title mol­ecule, C_13_H_13_NO, the dihedral angle between the benzene ring and the fused pyrrole ring is 2.03 (5)°. The methyl group at the 3-position has an equatorial orientation. The cyclo­hexene ring adopts an envelope conformation. Three C atoms of the cyclo­hexene ring, with their attached H atoms, and all atoms of the methyl group are disordered over two positions, the site-occupancy factors being 0.883 (2) and 0.117 (2). In the crystal structure, mol­ecules are stabilized by inter­molecular N—H⋯O hydrogen bonds. A C—H⋯π inter­action, involving the benzene ring, is also found.

## Related literature

For the biological activity of substituted 2,3,4,9-tetra­hydro­carbazoles, see: Mooradian *et al.* (1977 ▶); Jean *et al.* (2004 ▶); Biere *et al.* (1973 ▶); Lacoume (1973 ▶). For carbazole alkaloids, such as clausenapin, murrayafoline-A, murrayafoline-B, murrayastine, murrayaquinone-A, with a methyl substituent at the C-3 position, see: Knolker & Reddy (2002 ▶). For the preparation of 1-oxo compounds *via* their corresponding hydrazones, see: Sowmithran & Rajendra Prasad (1986 ▶); Rajendra Prasad & Vijayalakshmi (1994 ▶); Gunaseelan *et al.* (2007*a* ▶,*b* ▶); Sridharan *et al.* (2008 ▶); Thiruvalluvar *et al.* (2007 ▶).
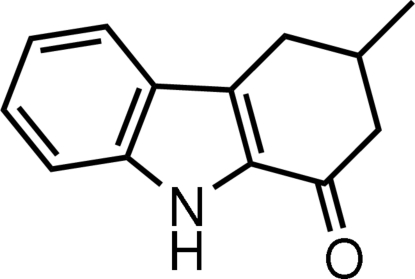


## Experimental

### 

#### Crystal data

C_13_H_13_NO
*M* *_r_* = 199.24
Triclinic, 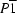

*a* = 5.8301 (3) Å
*b* = 8.4348 (5) Å
*c* = 10.8000 (7) Å
α = 78.094 (5)°
β = 75.942 (5)°
γ = 87.166 (5)°
*V* = 504.11 (5) Å^3^
*Z* = 2
Mo *K*α radiation
μ = 0.08 mm^−1^
*T* = 110 K
0.54 × 0.14 × 0.10 mm

#### Data collection

Oxford Diffraction Xcalibur Ruby diffractometer
Absorption correction: multi-scan (CrysAlis Pro; Oxford Diffraction, 2009 ▶) *T*_min_ = 0.753, *T*_max_ = 1.000 (expected range = 0.747–0.992)
5927 measured reflections
3292 independent reflections
2400 reflections with *I* > 2σ(*I*)
*R*_int_ = 0.028

#### Refinement

*R*[*F* ^2^ > 2σ(*F* ^2^)] = 0.053
*wR*(*F* ^2^) = 0.153
*S* = 1.00
3292 reflections
144 parameters
H atoms treated by a mixture of independent and constrained refinement
Δρ_max_ = 0.50 e Å^−3^
Δρ_min_ = −0.27 e Å^−3^

### 

Data collection: *CrysAlis Pro* (Oxford Diffraction, 2009 ▶); cell refinement: *CrysAlis Pro*; data reduction: *CrysAlis Pro*; program(s) used to solve structure: *SHELXS97* (Sheldrick, 2008 ▶); program(s) used to refine structure: *SHELXL97* (Sheldrick, 2008 ▶); molecular graphics: *ORTEP-3* (Farrugia, 1997 ▶); software used to prepare material for publication: *PLATON* (Spek, 2009 ▶).

## Supplementary Material

Crystal structure: contains datablocks global, I. DOI: 10.1107/S1600536809028050/wn2337sup1.cif

Structure factors: contains datablocks I. DOI: 10.1107/S1600536809028050/wn2337Isup2.hkl

Additional supplementary materials:  crystallographic information; 3D view; checkCIF report

## Figures and Tables

**Table 1:** Hydrogen-bond geometry (Å, °)

*D*—H⋯*A*	*D*—H	H⋯*A*	*D*⋯*A*	*D*—H⋯*A*
N9—H9⋯O1^i^	0.960 (17)	1.939 (16)	2.848 (1)	157.2 (13)
C4*A*—H4*B*⋯*Cg*1^ii^	0.99	2.83	3.779 (1)	162

Symmetry codes: (i) 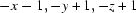; (ii) 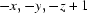. *Cg*1 is the centroid of the C4*D*,C5–C8,C8*A* ring.

**Fig. 1.**
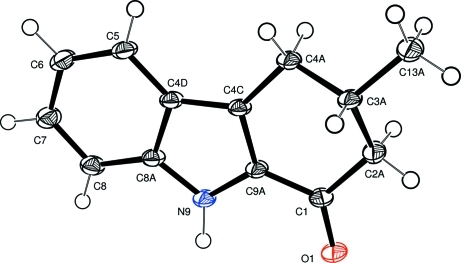
The molecular structure of the title compound, showing the atom-numbering scheme and displacement ellipsoids drawn at the 50% probability level. H atoms are shown as small spheres of arbitrary radius. Only the major disorder component is shown.

**Fig. 2.**
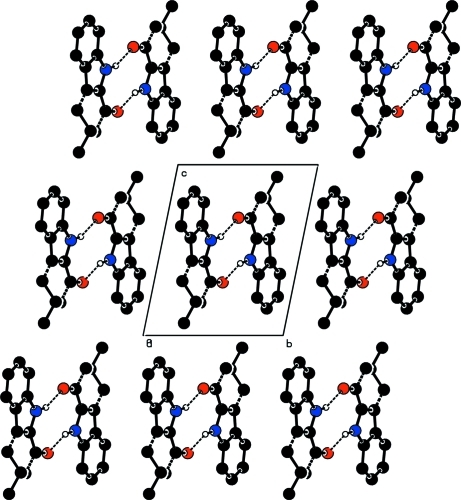
The molecular packing of the title compound, viewed down the a axis. Dashed lines indicate hydrogen bonds. H atoms not involved in hydrogen bonding have been omitted. Only the major disorder component is shown.

## supplementary crystallographic information

### Comment

Substituted 2,3,4,9-tetrahydrocarbazoles have been reported to possess many biological properties, such as central nervous system activity (Mooradian *et al.*, 1977), antihistamine (Jean *et al.*, 2004), antidiabetic (Biere *et al.*, 1973) and anti-inflammatory properties (Lacoume, 1973). We have attached importance to the title compound since some of the carbazole alkaloids, such as clausenapin, murrayafoline-A, murrayafoline-B, murrayastine, murrayaquinone-A have the methyl group as substituent at the C-3 position (Knolker & Reddy, 2002). The preparation of 1-oxo compounds *via* their corresponding hydrazones have been reported (Sowmithran & Rajendra Prasad, 1986; Rajendra Prasad & Vijayalakshmi, 1994). Guanaseelan *et al.* (2007*a*,*b*), Thiruvalluvar *et al.* (2007) and Sridharan *et al.* (2008) have reported the crystal structures of substituted carbazole derivatives, in which the carbazole units are not planar.

In the title molecule, C_13_H_13_NO, the dihedral angle between the benzene ring and the fused pyrrole ring is 2.03 (5)°. The methyl group at position 3 has an equatorial oreintation. The cyclohexene ring adopts an envelope conformation. In the crystal structure, the molecules are stabilized by intermolecular N9—H9···O1(-1 - *x*, 1 - *y*, 1 - *z*) hydrogen bonds. Furthermore, a C4A—H4B···π(-*x*, -*y*, 1 - *z*) interaction, involving the benzene ring(C4D—C8A), is also found in the crystal stucture.

### Experimental

A solution of 2-(2-phenylhydrazono)-5-methylcyclohexanone (0.216 g. 0.001 mol) in a mixture of acetic acid (20 ml) and hydrochloric acid (5 ml) was refluxed on an oil bath pre-heated to 398 K for 2 h. The contents were then cooled and poured into cold water with stirring. The brown solid which was separated by passing through a column of silica gel and eluted with a (98:2, *v*/*v*) petroleum ether-ethyl acetate mixture to yield the title compound (0.148 g, 74%). This was recrystallized from ethanol.

### Refinement

Atoms C2A, C3A, C4A of the cyclohexene ring, with attached hydrogen atoms, and all atoms of the methyl group are disordered over two positions; the site occupancy factors refined to 0.883 (2) and 0.117 (2). The H atom bonded to N9 was located in a difference Fourier map and refined isotropically. Other H atoms were positioned geometrically and allowed to ride on their parent atoms, with C—H = 0.95–1.00 Å and *U*_iso_(H) = *xU*_eq_(parent atom), where *x* = 1.5 for methyl and 1.2 for all other carbon-bound H atoms.

### Crystal data

**Table:**

C_13_H_13_NO	*Z* = 2
*M**_r_* = 199.24	*F*(000) = 212
Triclinic, *P*1	*D*_x_ = 1.313 Mg m^−^^3^
Hall symbol: -P 1	Melting point: 462 K
*a* = 5.8301 (3) Å	Mo *K*α radiation, λ = 0.71073 Å
*b* = 8.4348 (5) Å	Cell parameters from 2953 reflections
*c* = 10.8000 (7) Å	θ = 4.9–32.7°
α = 78.094 (5)°	µ = 0.08 mm^−^^1^
β = 75.942 (5)°	*T* = 110 K
γ = 87.166 (5)°	Needle, pale-yellow
*V* = 504.11 (5) Å^3^	0.54 × 0.14 × 0.10 mm

### Data collection

**Table:**

Oxford Diffraction Xcalibur Ruby diffractometer	3292 independent reflections
Radiation source: fine-focus sealed tube	2400 reflections with *I* > 2σ(*I*)
graphite	*R*_int_ = 0.028
Detector resolution: 10.5081 pixels mm^-1^	θ_max_ = 32.7°, θ_min_ = 4.9°
ω scans	*h* = −7→8
Absorption correction: multi-scan (*CrysAlis PRO*; Oxford Diffraction, 2009)	*k* = −10→12
*T*_min_ = 0.753, *T*_max_ = 1.000	*l* = −13→15
5927 measured reflections	

### Refinement

**Table:**

Refinement on *F*^2^	Primary atom site location: structure-invariant direct methods
Least-squares matrix: full	Secondary atom site location: difference Fourier map
*R*[*F*^2^ > 2σ(*F*^2^)] = 0.053	Hydrogen site location: inferred from neighbouring sites
*wR*(*F*^2^) = 0.153	H atoms treated by a mixture of independent and constrained refinement
*S* = 1.00	*w* = 1/[σ^2^(*F*_o_^2^) + (0.1015*P*)^2^] where *P* = (*F*_o_^2^ + 2*F*_c_^2^)/3
3292 reflections	(Δ/σ)_max_ = 0.001
144 parameters	Δρ_max_ = 0.50 e Å^−^^3^
0 restraints	Δρ_min_ = −0.27 e Å^−^^3^

### Special details

**Table:**

Geometry. Bond distances, angles *etc*. have been calculated using the rounded fractional coordinates. All su's are estimated from the variances of the (full) variance-covariance matrix. The cell e.s.d.'s are taken into account in the estimation of distances, angles and torsion angles
Refinement. Refinement of *F*^2^ against ALL reflections. The weighted *R*-factor *wR* and goodness of fit *S* are based on *F*^2^, conventional *R*-factors *R* are based on *F*, with *F* set to zero for negative *F*^2^. The threshold expression of *F*^2^ > 2σ(*F*^2^) is used only for calculating *R*-factors(gt) *etc*. and is not relevant to the choice of reflections for refinement. *R*-factors based on *F*^2^ are statistically about twice as large as those based on *F*, and *R*- factors based on ALL data will be even larger.

### Fractional atomic coordinates and isotropic or equivalent isotropic displacement parameters (Å^2^)

**Table:**

	*x*	*y*	*z*	*U*_iso_*/*U*_eq_	Occ. (<1)
O1	−0.36537 (13)	0.49067 (9)	0.31075 (7)	0.0255 (2)	
N9	−0.24180 (15)	0.34526 (10)	0.55707 (8)	0.0202 (2)	
C1	−0.18847 (17)	0.40400 (12)	0.31405 (9)	0.0202 (2)	
C2A	−0.03045 (19)	0.36084 (14)	0.19199 (10)	0.0265 (3)	0.883 (2)
C3A	0.22713 (19)	0.32688 (14)	0.19735 (10)	0.0199 (3)	0.883 (2)
C4A	0.24963 (17)	0.20104 (12)	0.31810 (9)	0.0202 (2)	0.883 (2)
C4C	0.08447 (16)	0.24013 (11)	0.43740 (9)	0.0179 (2)	
C4D	0.09067 (17)	0.19671 (11)	0.57178 (9)	0.0189 (2)	
C5	0.25223 (18)	0.11247 (12)	0.63950 (10)	0.0237 (3)	
C6	0.2021 (2)	0.09443 (13)	0.77313 (10)	0.0279 (3)	
C7	−0.0090 (2)	0.15661 (13)	0.84233 (10)	0.0275 (3)	
C8	−0.17079 (19)	0.24034 (12)	0.77927 (10)	0.0237 (3)	
C8A	−0.11770 (17)	0.26304 (11)	0.64282 (9)	0.0190 (2)	
C9A	−0.11878 (16)	0.33125 (11)	0.43268 (9)	0.0184 (2)	
C13A	0.3667 (2)	0.27429 (16)	0.07206 (11)	0.0331 (3)	0.883 (2)
C4B	0.24963 (17)	0.20104 (12)	0.31810 (9)	0.0202 (2)	0.117 (2)
C13B	0.3667 (2)	0.27429 (16)	0.07206 (11)	0.0331 (3)	0.117 (2)
C2B	−0.03045 (19)	0.36084 (14)	0.19199 (10)	0.0265 (3)	0.117 (2)
C3B	0.1473 (15)	0.2369 (11)	0.1984 (8)	0.0199 (3)	0.117 (2)
H3A	0.29806	0.43037	0.20208	0.0239*	0.883 (2)
H5	0.39331	0.06882	0.59392	0.0285*	
H4A	0.41424	0.19960	0.32791	0.0243*	0.883 (2)
H4B	0.21248	0.09211	0.30741	0.0243*	0.883 (2)
H8	−0.31286	0.28115	0.82635	0.0285*	
H9	−0.379 (3)	0.4111 (17)	0.5785 (14)	0.041 (4)*	
H13A	0.35221	0.35751	−0.00372	0.0496*	0.883 (2)
H13B	0.53365	0.26031	0.07418	0.0496*	0.883 (2)
H13C	0.30307	0.17155	0.06591	0.0496*	0.883 (2)
H6	0.31134	0.03927	0.81953	0.0335*	
H7	−0.04011	0.14027	0.93458	0.0329*	
H2A	−0.09634	0.26383	0.17480	0.0318*	0.883 (2)
H2B	−0.03456	0.45104	0.11761	0.0318*	0.883 (2)
H2C	0.05134	0.46162	0.13969	0.0318*	0.117 (2)
H2D	−0.13550	0.32986	0.14135	0.0318*	0.117 (2)
H3B	0.07301	0.13410	0.19484	0.0239*	0.117 (2)
H4C	0.39691	0.26440	0.29933	0.0243*	0.117 (2)
H4D	0.29160	0.08475	0.33572	0.0243*	0.117 (2)
H13D	0.30671	0.29814	−0.00707	0.0496*	0.117 (2)
H13E	0.45547	0.36781	0.07648	0.0496*	0.117 (2)
H13F	0.47089	0.17958	0.07016	0.0496*	0.117 (2)

### Atomic displacement parameters (Å^2^)

**Table:**

	*U*^11^	*U*^22^	*U*^33^	*U*^12^	*U*^13^	*U*^23^
O1	0.0174 (3)	0.0308 (4)	0.0286 (4)	0.0122 (3)	−0.0071 (3)	−0.0076 (3)
N9	0.0155 (3)	0.0238 (4)	0.0195 (4)	0.0088 (3)	−0.0018 (3)	−0.0049 (3)
C1	0.0136 (4)	0.0234 (4)	0.0237 (4)	0.0058 (3)	−0.0045 (3)	−0.0065 (3)
C2A	0.0206 (5)	0.0381 (6)	0.0213 (4)	0.0140 (4)	−0.0068 (3)	−0.0085 (4)
C3A	0.0151 (5)	0.0232 (5)	0.0196 (5)	0.0072 (4)	−0.0028 (4)	−0.0034 (4)
C4A	0.0146 (4)	0.0234 (4)	0.0213 (4)	0.0075 (3)	−0.0025 (3)	−0.0052 (3)
C4C	0.0137 (4)	0.0182 (4)	0.0200 (4)	0.0056 (3)	−0.0018 (3)	−0.0039 (3)
C4D	0.0160 (4)	0.0182 (4)	0.0207 (4)	0.0056 (3)	−0.0021 (3)	−0.0037 (3)
C5	0.0204 (5)	0.0239 (5)	0.0247 (5)	0.0107 (4)	−0.0045 (4)	−0.0036 (4)
C6	0.0289 (5)	0.0291 (5)	0.0240 (5)	0.0128 (4)	−0.0087 (4)	−0.0018 (4)
C7	0.0318 (5)	0.0266 (5)	0.0205 (5)	0.0095 (4)	−0.0042 (4)	−0.0016 (4)
C8	0.0236 (5)	0.0231 (4)	0.0209 (4)	0.0064 (4)	0.0000 (3)	−0.0041 (4)
C8A	0.0165 (4)	0.0176 (4)	0.0209 (4)	0.0051 (3)	−0.0021 (3)	−0.0033 (3)
C9A	0.0135 (4)	0.0211 (4)	0.0195 (4)	0.0063 (3)	−0.0028 (3)	−0.0042 (3)
C13A	0.0254 (5)	0.0475 (7)	0.0212 (5)	0.0185 (5)	−0.0010 (4)	−0.0047 (4)
C4B	0.0146 (4)	0.0234 (4)	0.0213 (4)	0.0075 (3)	−0.0025 (3)	−0.0052 (3)
C13B	0.0254 (5)	0.0475 (7)	0.0212 (5)	0.0185 (5)	−0.0010 (4)	−0.0047 (4)
C2B	0.0206 (5)	0.0381 (6)	0.0213 (4)	0.0140 (4)	−0.0068 (3)	−0.0085 (4)
C3B	0.0151 (5)	0.0232 (5)	0.0196 (5)	0.0072 (4)	−0.0028 (4)	−0.0034 (4)

### Geometric parameters (Å, °)

**Table:**

O1—C1	1.2377 (13)	C8—C8A	1.4043 (14)
N9—C8A	1.3686 (13)	C2A—H2A	0.9900
N9—C9A	1.3864 (12)	C2A—H2B	0.9900
N9—H9	0.960 (17)	C2B—H2C	0.9900
C1—C2A	1.5146 (14)	C2B—H2D	0.9900
C1—C2B	1.5146 (14)	C3A—H3A	1.0000
C1—C9A	1.4446 (13)	C3B—H3B	1.0000
C2A—C3A	1.5276 (16)	C4A—H4A	0.9900
C2B—C3B	1.439 (9)	C4A—H4B	0.9900
C3A—C4A	1.5306 (15)	C4B—H4C	0.9900
C3A—C13A	1.5385 (16)	C4B—H4D	0.9900
C3B—C13B	1.616 (9)	C5—H5	0.9500
C3B—C4B	1.521 (9)	C6—H6	0.9500
C4A—C4C	1.4957 (13)	C7—H7	0.9500
C4B—C4C	1.4957 (13)	C8—H8	0.9500
C4C—C4D	1.4306 (13)	C13A—H13B	0.9800
C4C—C9A	1.3859 (14)	C13A—H13C	0.9800
C4D—C5	1.4075 (15)	C13A—H13A	0.9800
C4D—C8A	1.4264 (14)	C13B—H13D	0.9800
C5—C6	1.3785 (15)	C13B—H13E	0.9800
C6—C7	1.4140 (16)	C13B—H13F	0.9800
C7—C8	1.3784 (16)		
			
O1···N9	2.9322 (11)	H3A···O1^iv^	2.6400
O1···C3A^i^	3.3896 (14)	H3A···C9A	3.0300
O1···C4A^i^	3.3747 (13)	H3A···C8^iii^	2.8700
O1···C4B^i^	3.3747 (13)	H3A···H8^iii^	2.3900
O1···N9^ii^	2.8481 (12)	H3B···C7^v^	2.6200
O1···H4A^i^	2.7800	H3B···C13A	2.1300
O1···H3A^i^	2.6400	H3B···C2A	1.9700
O1···H4C^i^	2.4500	H3B···C4A	2.0500
O1···H9	2.813 (15)	H3B···C5^v^	2.8900
O1···H9^ii^	1.939 (16)	H3B···C6^v^	2.4800
N9···O1	2.9322 (11)	H4A···C1^iv^	2.9100
N9···O1^ii^	2.8481 (12)	H4A···O1^iv^	2.7800
C1···C8A^iii^	3.5679 (14)	H4A···H5^viii^	2.5500
C1···C8^iii^	3.5708 (15)	H4B···C4D^v^	2.9600
C2B···C13A	2.5175 (17)	H4B···H13C	2.4900
C2B···C4A	2.5384 (15)	H4B···C8A^v^	2.9900
C3A···O1^iv^	3.3896 (14)	H4C···O1^iv^	2.4500
C3B···C6^v^	3.458 (9)	H4C···C1^iv^	2.7900
C3B···C7^v^	3.592 (9)	H4C···C2A	3.0000
C4A···O1^iv^	3.3747 (13)	H4C···C13A	2.4900
C4B···C2A	2.5384 (15)	H4C···H13E	2.3300
C4B···O1^iv^	3.3747 (13)	H4D···H5^viii^	2.3800
C4B···C13A	2.5282 (15)	H4D···C8A^v^	3.1000
C6···C3B^v^	3.458 (9)	H4D···C13A	2.9100
C7···C3B^v^	3.592 (9)	H5···H4D^viii^	2.3800
C8···C1^iii^	3.5708 (15)	H5···H4A^viii^	2.5500
C8A···C1^iii^	3.5679 (14)	H5···H5^viii^	2.5700
C13B···C2A	2.5175 (17)	H6···H13F^viii^	2.4600
C13B···C4A	2.5282 (15)	H7···H7^ix^	2.5800
C1···H4C^i^	2.7900	H8···H13D^x^	2.5100
C1···H9^ii^	3.012 (16)	H8···C13A^x^	2.8400
C1···H4A^i^	2.9100	H8···H13A^x^	2.4900
C4D···H4B^v^	2.9600	H8···H13B^x^	2.5800
C5···H3B^v^	2.8900	H8···C13B^x^	2.8400
C6···H2A^v^	3.0200	H8···H3A^iii^	2.3900
C6···H3B^v^	2.4800	H9···O1^ii^	1.939 (16)
C7···H3B^v^	2.6200	H9···O1	2.813 (15)
C8···H2C^iii^	2.9900	H9···C1^ii^	3.012 (16)
C8···H3A^iii^	2.8700	H13A···H8^vi^	2.4900
C8A···H4B^v^	2.9900	H13A···H2B	2.5000
C8A···H4D^v^	3.1000	H13B···H8^vi^	2.5800
C9A···H3A	3.0300	H13C···H4B	2.4900
C13A···H8^vi^	2.8400	H13C···H2A	2.5100
C13B···H8^vi^	2.8400	H13D···H8^vi^	2.5100
H2A···H13C	2.5100	H13D···C2A	2.6600
H2A···C6^v^	3.0200	H13D···H2C	2.4900
H2B···H2B^vii^	2.4400	H13E···C2A	2.8000
H2B···H13A	2.5000	H13E···C4A	2.7100
H2C···C13A	2.4400	H13E···H2C	2.4300
H2C···H13D	2.4900	H13E···H4C	2.3300
H2C···C8^iii^	2.9900	H13E···H13E^xi^	2.4800
H2C···H13E	2.4300	H13F···C4A	2.7100
H2C···C4A	2.9900	H13F···H6^viii^	2.4600
H2D···C13A	2.8800		
			
C8A—N9—C9A	107.71 (8)	C1—C2B—H2C	107.00
C9A—N9—H9	126.1 (9)	C1—C2B—H2D	107.00
C8A—N9—H9	125.8 (9)	C3B—C2B—H2C	107.00
O1—C1—C9A	123.78 (9)	C3B—C2B—H2D	107.00
O1—C1—C2A	121.93 (9)	H2C—C2B—H2D	107.00
C2B—C1—C9A	114.27 (9)	C4A—C3A—H3A	108.00
C2A—C1—C9A	114.27 (9)	C13A—C3A—H3A	108.00
O1—C1—C2B	121.93 (9)	C2A—C3A—H3A	108.00
C1—C2A—C3A	114.98 (9)	C2B—C3B—H3B	107.00
C1—C2B—C3B	121.6 (3)	C4B—C3B—H3B	107.00
C2A—C3A—C13A	110.39 (9)	C13B—C3B—H3B	107.00
C2A—C3A—C4A	112.21 (9)	C3A—C4A—H4A	110.00
C4A—C3A—C13A	110.93 (9)	C4C—C4A—H4A	110.00
C4B—C3B—C13B	107.3 (5)	C4C—C4A—H4B	110.00
C2B—C3B—C4B	118.1 (6)	C3A—C4A—H4B	110.00
C2B—C3B—C13B	110.9 (5)	H4A—C4A—H4B	108.00
C3A—C4A—C4C	110.43 (8)	C4C—C4B—H4D	109.00
C3B—C4B—C4C	113.5 (3)	C3B—C4B—H4C	109.00
C4A—C4C—C4D	131.07 (9)	C3B—C4B—H4D	109.00
C4A—C4C—C9A	122.53 (8)	H4C—C4B—H4D	108.00
C4D—C4C—C9A	106.40 (8)	C4C—C4B—H4C	109.00
C4B—C4C—C4D	131.07 (9)	C4D—C5—H5	121.00
C4B—C4C—C9A	122.53 (8)	C6—C5—H5	121.00
C4C—C4D—C5	134.36 (9)	C7—C6—H6	119.00
C4C—C4D—C8A	106.39 (8)	C5—C6—H6	119.00
C5—C4D—C8A	119.23 (9)	C8—C7—H7	119.00
C4D—C5—C6	118.82 (10)	C6—C7—H7	119.00
C5—C6—C7	121.15 (10)	C8A—C8—H8	121.00
C6—C7—C8	121.64 (10)	C7—C8—H8	121.00
C7—C8—C8A	117.49 (10)	H13B—C13A—H13C	109.00
N9—C8A—C4D	109.02 (8)	H13A—C13A—H13C	109.00
N9—C8A—C8	129.38 (9)	C3A—C13A—H13A	109.00
C4D—C8A—C8	121.61 (9)	C3A—C13A—H13B	109.00
N9—C9A—C1	125.11 (9)	C3A—C13A—H13C	109.00
N9—C9A—C4C	110.47 (8)	H13A—C13A—H13B	109.00
C1—C9A—C4C	124.40 (9)	C3B—C13B—H13D	109.00
C1—C2A—H2A	109.00	C3B—C13B—H13E	109.00
C1—C2A—H2B	109.00	C3B—C13B—H13F	109.00
C3A—C2A—H2A	109.00	H13D—C13B—H13E	109.00
C3A—C2A—H2B	109.00	H13D—C13B—H13F	109.00
H2A—C2A—H2B	108.00	H13E—C13B—H13F	109.00
			
C9A—N9—C8A—C4D	0.74 (11)	C9A—C4C—C4D—C5	−176.89 (11)
C9A—N9—C8A—C8	−179.21 (10)	C9A—C4C—C4D—C8A	1.25 (11)
C8A—N9—C9A—C1	−178.64 (9)	C4A—C4C—C9A—N9	178.40 (9)
C8A—N9—C9A—C4C	0.06 (12)	C4A—C4C—C9A—C1	−2.88 (15)
O1—C1—C2A—C3A	152.13 (10)	C4D—C4C—C9A—N9	−0.84 (11)
C9A—C1—C2A—C3A	−29.84 (13)	C4D—C4C—C9A—C1	177.89 (9)
O1—C1—C9A—N9	1.63 (16)	C4C—C4D—C5—C6	178.95 (11)
O1—C1—C9A—C4C	−176.91 (10)	C8A—C4D—C5—C6	1.00 (15)
C2A—C1—C9A—N9	−176.36 (9)	C4C—C4D—C8A—N9	−1.24 (11)
C2A—C1—C9A—C4C	5.10 (14)	C4C—C4D—C8A—C8	178.71 (9)
C1—C2A—C3A—C4A	52.21 (13)	C5—C4D—C8A—N9	177.23 (9)
C1—C2A—C3A—C13A	176.50 (10)	C5—C4D—C8A—C8	−2.82 (15)
C2A—C3A—C4A—C4C	−47.27 (12)	C4D—C5—C6—C7	1.00 (16)
C13A—C3A—C4A—C4C	−171.26 (9)	C5—C6—C7—C8	−1.31 (17)
C3A—C4A—C4C—C4D	−156.71 (10)	C6—C7—C8—C8A	−0.46 (16)
C3A—C4A—C4C—C9A	24.26 (13)	C7—C8—C8A—N9	−177.55 (10)
C4A—C4C—C4D—C5	3.97 (19)	C7—C8—C8A—C4D	2.50 (15)
C4A—C4C—C4D—C8A	−177.90 (10)		

Symmetry codes: (i) *x*−1, *y*, *z*; (ii) −*x*−1, −*y*+1, −*z*+1; (iii) −*x*, −*y*+1, −*z*+1; (iv) *x*+1, *y*, *z*; (v) −*x*, −*y*, −*z*+1; (vi) *x*+1, *y*, *z*−1; (vii) −*x*, −*y*+1, −*z*; (viii) −*x*+1, −*y*, −*z*+1; (ix) −*x*, −*y*, −*z*+2; (x) *x*−1, *y*, *z*+1; (xi) −*x*+1, −*y*+1, −*z*.

### Hydrogen-bond geometry (Å, °)

**Table:**

*D*—H···*A*	*D*—H	H···*A*	*D*···*A*	*D*—H···*A*
N9—H9···O1^ii^	0.960 (17)	1.939 (16)	2.848 (1)	157.2 (13)
C4A—H4B···Cg1^v^	0.99	2.83	3.779 (1)	162

Symmetry codes: (ii) −*x*−1, −*y*+1, −*z*+1; (v) −*x*, −*y*, −*z*+1.
